# A fractional-order mathematical model for COVID-19 outbreak with the effect of symptomatic and asymptomatic transmissions

**DOI:** 10.1140/epjp/s13360-022-02603-z

**Published:** 2022-03-28

**Authors:** Zeeshan Ali, Faranak Rabiei, Mohammad M. Rashidi, Touraj Khodadadi

**Affiliations:** 1 School of Engineering, Monash University Malaysia, 47500 Subang Jaya, Selangor Malaysia; 2 Institute of Fundamental and Frontier Sciences, University of Electronic Science and Technology of China, Chengdu, 610054 Sichuan People’s Republic of China; 3 Department of Information Technology, Malaysia University of Science and Technology, 47810 Petaling Jaya, Selangor Malaysia

## Abstract

The purpose of this paper is to investigate the transmission dynamics of a fractional-order mathematical model of COVID-19 including susceptible ($\textsc {S}$), exposed ($\textsc {E}$), asymptomatic infected ($\textsc {I}_1$), symptomatic infected ($\textsc {I}_2$), and recovered ($\textsc {R}$) classes named $\mathrm {SEI_{1}I_{2}R}$ model, using the Caputo fractional derivative. Here, $\mathrm {SEI_{1}I_{2}R}$ model describes the effect of asymptomatic and symptomatic transmissions on coronavirus disease outbreak. The existence and uniqueness of the solution are studied with the help of Schaefer- and Banach-type fixed point theorems. Sensitivity analysis of the model in terms of the variance of each parameter is examined, and the basic reproduction number $(R_{0})$ to discuss the local stability at two equilibrium points is proposed. Using the Routh–Hurwitz criterion of stability, it is found that the disease-free equilibrium will be stable for $R_{0} < 1$ whereas the endemic equilibrium becomes stable for $R_{0} > 1$ and unstable otherwise. Moreover, the numerical simulations for various values of fractional-order are carried out with the help of the fractional Euler method. The numerical results show that asymptomatic transmission has a lower impact on the disease outbreak rather than symptomatic transmission. Finally, the simulated graph of total infected population by proposed model here is compared with the real data of second-wave infected population of COVID-19 outbreak in India.

## Introduction

Mathematical models are powerful tools for understanding and preventing the infectious disease transmission. The researchers gain valuable information for several contagious diseases by studying stochastic and deterministic models. Kermack and McKendrick (1927) created a helpful model for executing and growing complex epidemic models, now regarded as a fundamental model in an epidemiology study [1]. Many infectious diseases can transmit in both vertical and horizontal paths. HIV/AIDS, Herpes Simplex, Hepatitis B, and Rubella are a few examples of such infections. These diseases are horizontally transmitted in people and animals by host-to-host interaction or disease carriers such as flies, mosquitoes, and others. The reader can find several good papers on the infectious disease modeling in the literature, for example, HIV/AIDS [2], Zika [3], Ebola [4], West Nile [5], Influenza [6], Dengue [7], Oncolytic [8], Cancer [9].

The first epidemic of severe acute respiratory syndrome (SARS) occurred in mainland China in 2003, while another outbreak is known as Middle East respiratory syndrome (MERS) occurred in South Korea in 2015. The new virus (2019-nCoV), which is highly transmissible and virulent, was identified in a single individual in the Chinese city of Wuhan [10]. This new disease, which produces a severe acute respiratory syndrome, has spread throughout the world. There have been over 253 million confirmed COVID-19 registered cases, and over 5.1 million fatalities globally since November 10, 2021 [11]. America, Europe, Africa, Southeast Asia, the Western Pacific, the Eastern Pacific, and the Mediterranean are the most effected by the coronavirus. The first signs of a COVID-19 infection include a dry cough, fever, exhaustion, and shortness of breath, which develop in 2–10 days and can lead to pneumonia, SARS, kidney failure, and even death [12]. The pandemic continued to expand, but vaccinations have now halted the transmission. Many researchers have been developed mathematical models for the transmission of the aforesaid disease involves various compartments, for example Susceptible-Infected (SI) [13], SI-Removed (SIR) [14], S-Exposed-IR (SEIR) [15], SEI-Quarantined-R (SEIQR) [16], and SIQ-Home Quarantined-R-Diseased (SIQHRD) [17].

For a year, there were no particular treatment approaches such as antibiotics or other treatments against the coronavirus because it is a single RNA virus. As a result, non-pharmaceutical measures such as isolation, social distance, quarantine, awareness programs, and personal hygiene, such as wearing a mask and washing hands regularly, become more helpful in reducing COVID-19 disease transmission. Interestingly, it has been observed that individuals who take all precautionary measures are occasionally infected, making it necessary to investigate the facts in human-to-human transmission processes. Even though nothing is known clinically about the COVID-19 transmission pathway, numerous compartmental models offer simulation graphs that are quite similar to statistical data. In most situations, asymptomatic people are excluded from the COVID-19 diagnostic test and play a substantial role in the disease’s transmission. There have also been several reports of other diseases’ deaths yet later tested positive with COVID-19. Because COVID-19 is a lot more infectious disease than being deadly, a massive proportion of the population eventually becomes a carrier. To address all of these factors, no mathematical model for parallel disease transmission has been developed on COVID-19, and hence a mathematical model for symptomatic and asymptomatic disease transmission at the same time is required. Consequently, the goal of this research is to provide a mathematical model for controlling the COVID-19 epidemic. The new mathematical model, known as $\mathrm {SEI_{1}I_{2}R}$ model, considers two parallel paths for symptomatic and asymptomatic transmission throughout the exposed compartment. The model examines the COVID-19 outbreak’s asymptomatic transmission impact as well as the sensitivity of the parameters affecting the pandemic.

The function representing the disease’s transmission mechanism, known as the force of infection or incidence rate, plays a crucial role in SIR, SEIR, and similar models. Typically, such a function is dependent on both the susceptible and infective classes, for example $f(\textsc {S},\textsc {I})$. The incidence rate can be expressed in a simple way using the mass action principle: If $\mu$ is the per capita contact rate, then the infection is assumed to spread with the rate $f(\textsc {S},\textsc {I}) = \mu \textsc {S}\textsc {I}$. Capasso and his colleagues [18] have emphasized the need to consider nonlinear incidence rates for some specific diseases since the 1970s: The studied example was the spread of cholera epidemic. Many researchers have suggested different nonlinear forms for the incidence rate since then, see [19]. The functional $f(\textsc {S},\textsc {I})=\mu \textsc {S}\textsc {I}(1+\lambda \textsc {I})$ is an excellent example of the nonlinear incidence rate, where the constants $\mu$ and $\lambda$ are both positive. It refers to a higher infection rate over a short period, which is caused by two exposures. The contact rate $\mu \textsc {S}\textsc {I}$ is the result of single contact, while the term $\mu \lambda \textsc {I}^{2}\textsc {S}$ refers to a new infective that arises as a result of the double exposure [20]. The readers may see more detail about incident rate in [21]. The reason for choosing the convex incident rate is because this function could model the increase in infection resulting from the situation of double exposure. This is in line with the unknown disease mechanisms of a COVID-19 pandemic studied in [22].

Over the last three decades, fractional derivatives have fascinated many researchers due to the recognition that, compared to classical derivatives, fractional derivatives are more efficient tools for modeling real-world phenomena. Fractional calculus (FC)-based modeling is becoming increasingly popular in dynamic situations. The fractional-order differential operators such as Riemann–Liouville (R-L) [23], Caputo [24], Erdélyi-Kober [25], Hilfer [26], Hadamard [27], Katugampola [28], Caputo–Fabrizio [29], and Atangana–Baleanu [30] change the ordinary model into a generalized model. This paper presents new research on a fractional-order dynamical model that underpins the spread of coronavirus infectious disease and can be used to predict its spread. So, in this manuscript, we develop a new mathematical model for COVID-19 outbreak and extend it to a fractional-order model by adding the Caputo sense of fractional derivative. The reason for utilizing the Caputo fractional derivative is that it possesses several fundamental characteristics of FC.

Furthermore, the Caputo operator may be useful to further specify the transmission behavior given in the model. Several previous investigations have found the reliability of the Caputo operator and their applicability to diverse the models arising in numerous areas of engineering and other sciences. A few related studies on Caputo and other fractional-order derivative can be found in the literature, for example physics and polymer technology [31], electrical circuits [32], electrochemistry [33], electrodynamics of complex medium [34], fluid mechanics [35], control theory [36], thermodynamics [37], neural network [38], image encryption [39], chaos [40], viscoelasticity [41], aerodynamics [42], capacitor theory [43], biology [44], blood flow [45], and the references cited therein. Besides, nowadays the researchers are devoting their research work to the fractional-order COVID-19 mathematical models. A huge number of good research papers related to fractional-order COVID-19 mathematical models can be found in the literature, some of them are the following: Caputo fractional-order [46, 47], Caputo–Fabrizio fractional-order [48, 49], Atangana–Baleanu fractional-order [50, 51], fuzzy fractional-order [52, 53], fractal-fractional order [54, 55].

The remaining part of the paper is carried out as follows: The COVID-19 model formulation is discussed in Sect. 2. Section 3 is devoted to basic FC tools and fundamental definitions, which will be used to determine theoretical outcomes. In Sect. 4, using the Schaefer- and Banach-type fixed point theorems, the appropriate conditions for existence and uniqueness are derived. In Sec. 5, the well-posedness and the biological feasibility of the model are obtained. Section 6 is devoted to the disease-free and endemic equilibrium point. The basic reproduction number ($R_{0}$) is obtained in Sect. 7, and the stability of the equilibrium points is proved in Sect. 8. In Sect. 9, the sensitivity analysis of $R_{0}$ is discussed. Sections 10, and 11 are devoted to numerical simulation/discussion and conclusion, respectively.

## Model formulation

A mathematical model is an abstract model that uses mathematical objects to explain the behavior of a real-life situation. Mathematical models may help in making better decisions about a particular process and studying functional relationships. Moreover, they may also be used to predict the quantitative behavior of a system. Due to the above importance of mathematical modeling, in this section, we developed an $\mathrm {SEI_{1}I_{2}R}$ model for COVID-19 disease transmission, in which the population is divided into five classes: susceptible $\textsc {S}$, exposed $\textsc {E}$, asymptomatic infected $\textsc {I}_{1}$, symptomatic infected $\textsc {I}_{2}$, and recovered or removed $\textsc {R}$. The model assumes the total population $\textsc {N}$ is not constant throughout the time and equal to the sum of all compartment sizes at any time $\mathrm {t}$, i.e., $\textsc {N}=\textsc {S}+\textsc {E}+\textsc {I}_{1}+\textsc {I}_{\textsc {2}}+\textsc {R}.$ We made the following assumptions to formulate the model: Asymptomatic transmission is represented by the primary pathway $\textsc {S} \textsc {E} \textsc {I}_{1} \textsc {R}$, in which asymptomatic infected individuals spread the disease at a rate of $\mu _{1}$. The $\textsc {S} \textsc {E} \textsc {I}_{2} \textsc {R}$ is the second pathway that denotes indicative transmission, in which symptomatic individuals transmit the infection at a rate of $\mu _{2}$. The symptomatic case’s transmission rate $\mu _{2}$ and infection rates $\rho _{2}$ are considered to be higher than the asymptomatic case’s, i.e., $\mu _{2}>\mu _{1}$ and $\rho _{2}>\rho _{1}$. Asymptomatic patients have a higher recovery rate than symptomatic patients $(\beta _{1}>\beta _{2})$, and asymptomatic patients have a lower mortality rate $(\alpha _{2}>\alpha _{1})$ than symptomatic patients. $\Pi$ is the recruitment rate, representing the increment of compartment $\textsc {S}$ in the form of birth or migration or recovered individuals. The natural death rate $\alpha$ is equal to the recruiting rate $\Pi$ into compartment $\textsc {S}$. In this study death population due to the COVID-19 infection is only counted.

1 $$\begin{aligned} \left\{ \begin{aligned}&\frac{\mathrm {d} \textsc {S}}{\mathrm {d} \mathrm {t}}=\frac{\Pi }{\textsc {N}}-\mu _{1}\textsc {S}\frac{\textsc {I}_{1}}{\textsc {N}}(1+\lambda _{1}\frac{\textsc {I}_{1}}{\textsc {N}})-\mu _{2} \textsc {S}\frac{\textsc {I}_{2}}{\textsc {N}}(1+\lambda _{2}\frac{\textsc {I}_{2}}{\textsc {N}})-\alpha \textsc {S}, \\&\frac{\mathrm {d} \textsc {E}}{\mathrm {d} \mathrm {t}}=\mu _{1}\textsc {S}\frac{\textsc {I}_{1}}{\textsc {N}}(1+\lambda _{1}\frac{\textsc {I}_{1}}{\textsc {N}})+\mu _{2} \textsc {S}\frac{\textsc {I}_{2}}{\textsc {N}}(1+\lambda _{2}\frac{\textsc {I}_{2}}{\textsc {N}})-(\rho _{1}+\rho _{2}) \textsc {E}, \\&\frac{\mathrm {d} \textsc {I}_{\textsc {1}}}{\mathrm {d} \mathrm {t}}=\rho _{1} \textsc {E}-(\alpha _{1}+\beta _{1}) \textsc {I}_{1}, \\&\frac{\mathrm {d} \textsc {I}_{2}}{\mathrm {d} \mathrm {t}}=\rho _{2} \textsc {E}-(\alpha _{2}+\beta _{2}) \textsc {I}_{2}, \\&\frac{\mathrm {d} \textsc {R}}{\mathrm {d} \mathrm {t}}=\beta _{1} \textsc {I}_{1}+\beta _{2} \textsc {I}_{2}-\alpha \textsc {R}, \end{aligned}\right. \end{aligned}$$

where $\mu _{\mathscr {i}}, \rho _{\mathscr {i}}, \beta _{\mathscr {i}}$, and $\alpha _{\mathscr {i}}(\mathscr {i}=1,2)$ denotes the transmission rate, infection rate, recovery rate, and death rate in the mentioned above pathways, respectively. $\lambda _{1}$ and $\lambda _{2}$ are the positive constants. The quantities $\mu _{1} \textsc {S} \frac{\textsc {I}_{1}}{\textsc {N}}$ and $\mu _{2} \textsc {S} \frac{\textsc {I}_{2}}{\textsc {N}}$ in the first equation represent the interaction of susceptible individuals with asymptomatic and symptomatic infectious peoples with $\mu _{1}$ and $\mu _{2}$ rates, respectively. As can be seen in the second equation, these interactions increase the number of people who are exposed. The terms $\rho _{1} \textsc {E}$ and $\rho _{2} \textsc {E}$ denote the number of people becoming infected via the exposure of susceptible people. In the third equation, $\alpha _{1} \textsc {I}_{1}$ and $\beta _{1} \textsc {I}_{1}$ show the number of death and recovered population in the asymptomatic class. The terms $\alpha _{2} \textsc {I}_{2}$ and $\beta _{2} \textsc {I}_{2}$ in the fourth equation specify the number of death and recovered individuals in symptomatic class. The term $\alpha \textsc {S}$ represents the natural death of susceptible people and $\alpha \textsc {R}$ shows the death of recovered people. Dividing all the equations of the model (1) by $(\textsc {N})$ and setting $\textsc {S}=\frac{\textsc {S}}{\textsc {N}}, \textsc {E}=\frac{\textsc {E}}{\textsc {N}}, \textsc {I}_{1}=\frac{\textsc {I}_{1}}{\textsc {N}}, \textsc {I}_{2}=\frac{\textsc {I}_{2}}{\textsc {N}}, \textsc {R}=\frac{\textsc {R}}{\textsc {N}}$ and $\Lambda =\frac{\Pi }{\textsc {N}}$ (recruitment rate), as a result, model (1) may be expressed as:

2 $$\begin{aligned} \left\{ \begin{aligned}&\frac{\mathrm {d} \textsc {S}}{\mathrm {d} \mathrm {t}}=\Lambda -\mu _{1}\textsc {S}\textsc {I}_{1}(1+\lambda _{1}\textsc {I}_{1})-\mu _{2}\textsc {S}\textsc {I}_{2}(1+\lambda _{2}\textsc {I}_{2})-\alpha \textsc {S}, \\&\frac{\mathrm {d} \textsc {E}}{\mathrm {d} \mathrm {t}}=\mu _{1}\textsc {S}\textsc {I}_{1}(1+\lambda _{1}\textsc {I}_{1})+\mu _{2}\textsc {S}\textsc {I}_{2}(1+\lambda _{2}\textsc {I}_{2})-(\rho _{1}+\rho _{2})\textsc {E}, \\&\frac{\mathrm {d} \textsc {I}_{1}}{\mathrm {d} \mathrm {t}}=\rho _{1} \textsc {E}-(\alpha _{1} +\beta _{1}) \textsc {I}_{1}, \\&\frac{\mathrm {d} \textsc {I}_{2}}{\mathrm {d} \mathrm {t}}=\rho _{2} \textsc {E}-(\alpha _{2} +\beta _{2} )\textsc {I}_{2}, \\&\frac{\mathrm {d} \textsc {R}}{\mathrm {d} \mathrm {t}}=\beta _{1} \textsc {I}_{1}+\beta _{2} \textsc {I}_{2}-\alpha \textsc {R}, \end{aligned}\right. \end{aligned}$$

where $\textsc {S}+\textsc {E}+\textsc {I}_{1}+\textsc {I}_{2}+\textsc {R}=1$. The proposed mathematical model for COVID-19 outbreak is considered under the initial conditions: $\textsc {S}(0), \textsc {E}(0), \textsc {I}_{1}(0), \textsc {I}_{2}(0), \textsc {R}(0) \ge 0$. All of the involved parameters $(\mu _{\mathscr {i}}, \lambda _{\mathscr {i}}, \rho _{\mathscr {i}}, \beta _{\mathscr {i}}, \alpha _{\mathscr {i}}),\;(\mathscr {i}=1,2)$ are considered to be nonnegative. The model is designed to look at the role of asymptomatic transmission rates in the COVID-19 epidemic in comparison to symptomatic transmission rates.

**Fig. 1:**
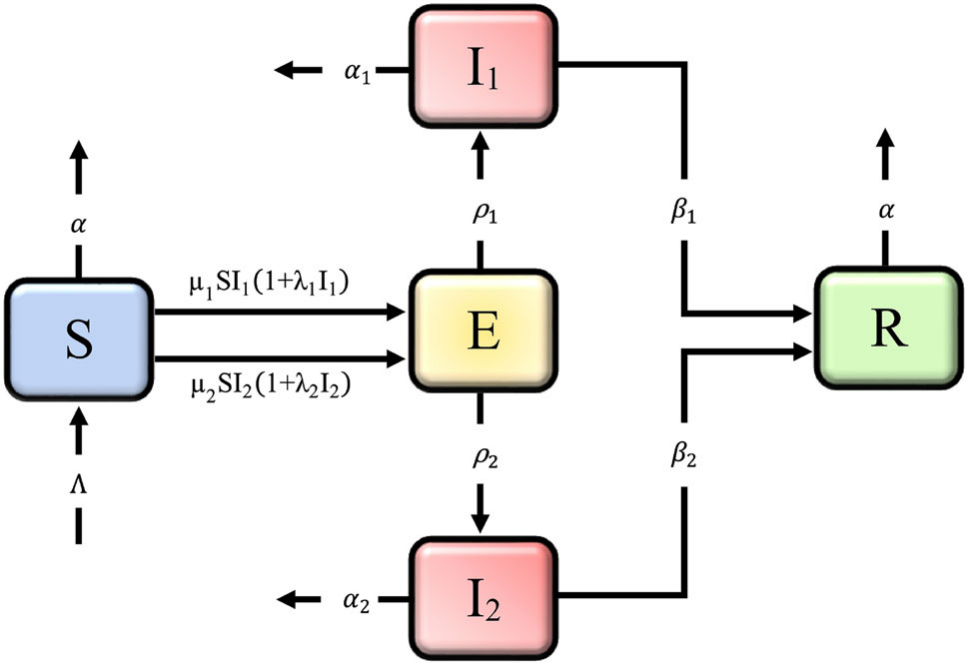
Flowchart of the proposed model (3)

Using the above assumptions, we propose the following $\mathrm {SEIR}$ compartmental model of order $0<\theta \le 1$ at time $\mathrm {t}\in [0,\mathrm {T}],\;\mathrm {T}<\infty$ as:

3 $$\begin{aligned} \left\{ \begin{aligned} ^{\mathrm {C}}{\mathbf {D}}^{\theta }\textsc {S}&=\Lambda -\mu _{1}\textsc {S}\textsc {I}_{1}(1+\lambda _{1}\textsc {I}_{1})-\mu _{2}\textsc {S}\textsc {I}_{2}(1+\lambda _{2}\textsc {I}_{2})-\alpha \textsc {S}, \\ ^{\mathrm {C}}{\mathbf {D}}^{\theta }\textsc {E}&=\mu _{1}\textsc {S}\textsc {I}_{1}(1+\lambda _{1}\textsc {I}_{1})+\mu _{2}\textsc {S}\textsc {I}_{2}(1+\lambda _{2}\textsc {I}_{2})-(\rho _{1}+\rho _{2})\textsc {E},\\ ^{\mathrm {C}}{\mathbf {D}}^{\theta }\textsc {I}_{1}&=\rho _{1}\textsc {E}-(\alpha _{1} +\beta _{1}) \textsc {I}_{1},\\ ^{\mathrm {C}}{\mathbf {D}}^{\theta }\textsc {I}_{2}&=\rho _{2}\textsc {E}-(\alpha _{2} +\beta _{2} )\textsc {I}_{2},\\ ^{\mathrm {C}}{\mathbf {D}}^{\theta }\textsc {R}&=\beta _{1}\textsc {I}_{1}+\beta _{2}\textsc {I}_{2}-\alpha \textsc {R}, \end{aligned}\right. \end{aligned}$$

subject to the following initial conditions

$$\begin{aligned} \textsc {S}(0),\;\textsc {E}(0),\;\textsc {I}_{1}(0),\;\textsc {I}_{2}(0),\;\textsc {R}(0)\ge 0. \end{aligned}$$

The flowchart for the proposed model (3) can be seen in Fig. 1.

## Preliminary definitions

This section is dedicated to some basic definitions and lemma that are necessary for achieving the existence theory.

### Definition 3.1

[27] The integral of order $\theta >0$ of a function  in the sense of R-L is the following:

### Definition 3.2

[27] For derivative of order $\theta >0$ of a function , in the sense of Caputo is the following:

### Lemma 3.1

[27] If $\theta >0$, then the differential equation  will have the following solution:where $\mathscr {n}=[\theta ]+1$.

## Existence theory of model (3)

We construct the existence theory for the considered model (3) in this section.

Let us write model (3) as follows:

4 $$\begin{aligned} \left\{ \begin{aligned} f_{1}(\mathrm {t},\textsc {S}, \textsc {E}, \textsc {I}_{1}, \textsc {I}_{2},\textsc {R})&=\Lambda -\mu _{1}\textsc {S}\textsc {I}_{1}(1+\lambda _{1}\textsc {I}_{1})-\mu _{2}\textsc {S}\textsc {I}_{2}(1+\lambda _{2}\textsc {I}_{2})-\alpha \textsc {S},\\ f_{2}(\mathrm {t},\textsc {S}, \textsc {E}, \textsc {I}_{1}, \textsc {I}_{2},\textsc {R})&=\mu _{1}\textsc {S}\textsc {I}_{1}(1+\lambda _{1}\textsc {I}_{1})+\mu _{2}\textsc {S}\textsc {I}_{2}(1+\lambda _{2}\textsc {I}_{2})-(\rho _{1}+\rho _{2})\textsc {E},\\ f_{3}(\mathrm {t},\textsc {S}, \textsc {E}, \textsc {I}_{1}, \textsc {I}_{2},\textsc {R})&=\rho _{1}\textsc {E}-(\alpha _{1}+\beta _{1})\textsc {I}_{1},\\ f_{4}(\mathrm {t},\textsc {S}, \textsc {E}, \textsc {I}_{1}, \textsc {I}_{2},\textsc {R})&=\rho _{2}\textsc {E}-(\alpha _{2}+\beta _{2})\textsc {I}_{2},\\ f_{5}(\mathrm {t},\textsc {S}, \textsc {E}, \textsc {I}_{1}, \textsc {I}_{2},\textsc {R})&=\beta _{1}\textsc {I}_{1}-\beta _{2}\textsc {I}_{2}-\alpha \textsc {R}. \end{aligned}\right. \end{aligned}$$

The proposed problem (3) can be reformulated in the following form:

5 $$\begin{aligned} \left\{ \begin{aligned} ^{\mathrm {C}}{\mathbf {D}}^{\theta }\textsc {S}&= f_{1}(\mathrm {t},\textsc {S}, \textsc {E}, \textsc {I}_{1}, \textsc {I}_{2},\textsc {R}), \\ ^{\mathrm {C}}{\mathbf {D}}^{\theta }\textsc {E}&=f_{2}(\mathrm {t},\textsc {S}, \textsc {E}, \textsc {I}_{1}, \textsc {I}_{2},\textsc {R}), \\ ^{\mathrm {C}}{\mathbf {D}}^{\theta }\textsc {I}_{1}&=f_{3}(\mathrm {t},\textsc {S}, \textsc {E}, \textsc {I}_{1}, \textsc {I}_{2},\textsc {R}),\\ ^{\mathrm {C}}{\mathbf {D}}^{\theta }\textsc {I}_{2}&=f_{4}(\mathrm {t},\textsc {S}, \textsc {E}, \textsc {I}_{1}, \textsc {I}_{2},\textsc {R}),\\ ^{\mathrm {C}}{\mathbf {D}}^{\theta }\textsc {R}&=f_{5}(\mathrm {t},\textsc {S}, \textsc {E}, \textsc {I}_{1}, \textsc {I}_{2},\textsc {R}). \end{aligned}\right. \end{aligned}$$

The considered system (3) can be written in the form presented using Eq. (4): 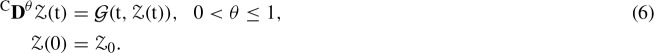 Equation (6) is solved using the R-L type integral as follows: 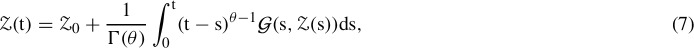 where 
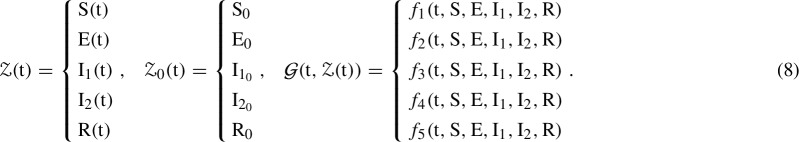
 For the existence theory, we define Banach space $\mathcal {J}=\mathbb {W}_{1}\times \mathbb {W}_{2}\times \mathbb {W}_{3}\times \mathbb {W}_{4}\times \mathbb {W}_{5}$, where $\mathbb {W}_{\mathscr {i}}=C([0,\mathrm {T}]),\;(\mathscr {i}=1,2,\dots ,5)$ under the norm .

Let $\mathcal {U}:\mathcal {J}\rightarrow \mathcal {J}$ be an operator defined as follows: 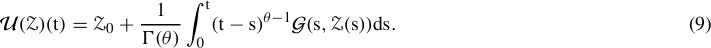


The existence of a solution is proved using the following theorem.

### Theorem 4.1

[56] Suppose $\mathcal {U}:\mathcal {J}\rightarrow \mathcal {J}$ is completely continuous and let  be bounded. Then $\mathcal {U}$ has at least one fixed point in $\mathcal {J}$.

### Theorem 4.2

Let $\mathcal {G}:[0,\mathrm {T}]\times \mathcal {J}\rightarrow \mathbb {R}$ is continuous and the following hypothesis:

($${\mathbf {P}}$$)
There is constant $\mathcal {L}_{\mathcal {G}}>0$ such that for , then

is hold. There is at least one solution for the considered system (3) .

### Proof

We begin by proving that the operator $\mathcal {U}$ is completely continuous. Suppose a sequence  such that  in $\mathcal {J},$ then for $\mathrm {t}\in [0,\mathrm {T}],$ we haveSince, , so  as $\mathscr {n}\rightarrow 0$. Thus $\mathcal {U}$ is continues. Let a bounded set $\mathcal {S}\subset \mathcal {J}$. Then by definition of $\mathcal {S}$,  Then for each  we can obtainThus, $\mathcal {U}$ is uniformly bounded.

Further, suppose $0\le \mathrm {t}_{2}\le \mathrm {t}_{1}\le \mathrm {T}.$ ThenAs a result, $\mathcal {U}$ is equicontinuous. Since $\mathcal {U}$ is both continuous and bounded, it is compact and so completely continuous. Let , we need to confirm that $\Phi$ is bounded. Suppose  then for $\mathrm {t}\in [0,\mathrm {T}],$ we have:Hence, the operator is completely continuous and the set $\Phi$ is bounded. Therefore, according to Theorem 4.1, $\mathcal {U}$ has at least one fixed point. As a consequence, the considered system (3) has the same number of solution. $\square$

To prove the uniqueness, we utilize the Banach’s fixed point theorem [56].

### Theorem 4.3

If the assumption $({\mathbf {P}})$ is hold and $\frac{\mathrm {T}^{\theta }\mathcal {L}_{{\mathcal {G}}}}{\Gamma (\theta +1)}<1$, then the solution of system (3) is unique.

### Proof

We define $\max _{\mathrm {t}\in [0,\mathrm {T}]}|\mathcal {G}(\mathrm {t},0)|=\mathcal {K}_{\mathcal {G}}<\infty ,$ such that $\mathcal {R}_{0}\ge \frac{\mathcal {K}_{{\mathcal {G}}}\Gamma (\theta +1)}{\Gamma (\theta +1)-\mathrm {T}^{\theta }\mathcal {L}_{{\mathcal {G}}}}$. We have to show that $\mathcal {U}(\mathcal {A}_{\mathcal {R}_{0}})\subset \mathcal {A}_{\mathcal {R}_{0}},$ where  For  we haveNow, in view of $({\mathbf {P}})$, for  and for each $\mathrm {t}\in [0,\mathrm {T}],$ we have 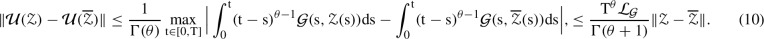 The contraction of $\mathcal {U}$ proves that the solution to the problem under consideration is unique. $\square$

## Well-posedness and biological feasibility

This section looks at the interval and region where our system’s solution will make perfect historical sense. Let $\textsc {N}$ be the net population size, i.e., $\textsc {N}(\mathrm {t})=\textsc {S}(\mathrm {t})+\textsc {E}(\mathrm {t})+\textsc {I}_{1}(\mathrm {t})+\textsc {I}_{2}(\mathrm {t})+\textsc {R}(\mathrm {t})$. Then

$$\begin{aligned} \textsc {N}^{'}(\mathrm {t})&=\Lambda -\alpha \textsc {S}-\alpha _{1}\textsc {I}_{1}-\alpha _{2}\textsc {I}_{2}-\alpha \textsc {R},\\&\le \Lambda -\alpha \textsc {R}. \end{aligned}$$

Since, $\textsc {R}\le \textsc {N}$. So the above inequality becomes:

$$\begin{aligned} \textsc {N}^{'}(\mathrm {t})&\le \Lambda -\alpha \textsc {N}. \end{aligned}$$

We want that the function $\textsc {N}(\mathrm {t})$ is a positively increasing function $\textsc {N}^{'}(\mathrm {t})>0$ then $\textsc {N}(\mathrm {t})< \frac{\Lambda }{\alpha }$ and $\textsc {N}^{'}(\mathrm {t})\ge \textsc {N}(\mathrm {t})$. The aforementioned inequality is known as the threshold population level in the literature. As a result, we infer that the accepted set of solutions to the proposed model must be contained within:

11 $$\begin{aligned} \Theta =\Big \{(\textsc {S}, \textsc {E}, \textsc {I}_{1}, \textsc {I}_{2}, \textsc {R})\in \mathbb {R}_{+}^{5}:0\le \textsc {S}+\textsc {E}+\textsc {I}_{1}+\textsc {I}_{2}+\textsc {R}<\frac{\Lambda }{\alpha }\Big \} \end{aligned}$$

Here in biological terms $\mathbb {R}_{+}^{5}$ is the positive cone of $\mathbb {R}^{5}$ that also contains its lower-dimensional faces.

## Disease-free and endemic equilibrium point

### Disease-free equilibrium point

The point that no disease exists in the population is known as the disease-free equilibrium point. Let set $^{\mathrm {C}}{\mathbf {D}}^{\theta }\textsc {S}(\mathrm {t})=^{\mathrm {C}}{\mathbf {D}}^{\theta }\textsc {E}(\mathrm {t})=^{\mathrm {C}}{\mathbf {D}}^{\theta }\textsc {I}_{1}(\mathrm {t})=^{\mathrm {C}}{\mathbf {D}}^{\theta }\textsc {I}_{2}(\mathrm {t})=^{\mathrm {C}}{\mathbf {D}}^{\theta }\textsc {R}(\mathrm {t})=0$ to compute the equilibrium points from system (3). Then

12 $$\begin{aligned} \left\{ \begin{aligned}&\Lambda -\mu _{1}\textsc {S}(\mathrm {t})\textsc {I}_{1}(\mathrm {t})(1+\lambda _{1}\textsc {I}_{1}(\mathrm {t}))-\mu _{2}\textsc {S}(\mathrm {t})\textsc {I}_{2}(\mathrm {t})(1+\lambda _{2}\textsc {I}_{2}(\mathrm {t}))-\alpha \textsc {S}(\mathrm {t})=0, \\ {}&\mu _{1}\textsc {S}(\mathrm {t})\textsc {I}_{1}(\mathrm {t})(1+\lambda _{1}\textsc {I}_{1}(\mathrm {t}))+\mu _{2}\textsc {S}(\mathrm {t})\textsc {I}_{2}(\mathrm {t})(1+\lambda _{2}\textsc {I}_{2}(\mathrm {t}))-(\rho _{1}+\rho _{2})\textsc {E}(\mathrm {t})=0, \\ {}&\rho _{1}\textsc {E}(\mathrm {t})-(\alpha _{1}+\beta _{1})\textsc {I}_{1}(\mathrm {t})=0,\\ {}&\rho _{2}\textsc {E}(\mathrm {t})-(\alpha _{2}+\beta _{2})\textsc {I}_{2}(\mathrm {t})=0,\\ {}&\beta _{1}\textsc {I}_{1}(\mathrm {t})-\beta _{2}\textsc {I}_{2}(\mathrm {t})-\alpha \textsc {R}(\mathrm {t})=0.&\end{aligned}\right. \end{aligned}$$

Applying the required conditions $\textsc {E}=\textsc {I}_{1}=\textsc {I}_{2}=0$ in the system (12) for disease-free equilibrium, then the disease-free equilibrium point of the considered model (3) is given below:

13 $$\begin{aligned} (\textsc {S}^{0}, \textsc {E}^{0}, \textsc {I}_{1}^{0}, \textsc {I}_{2}^{0}, \textsc {R}^{0})=\left( \frac{\Lambda }{\alpha }, 0, 0, 0, 0\right) . \end{aligned}$$

### Endemic equilibrium point

The endemic equilibrium point $(\textsc {S}^{*}, \textsc {E}^{*}, \textsc {I}_{1}^{*}, \textsc {I}_{2}^{*}, \textsc {R}^{*})$ of the system (12) is as follows:

$$\begin{aligned}&\textsc {S}^{*}=\frac{\Lambda }{\alpha +\mu _1 \textsc {I}_{1}^2 \lambda _1+\mu _2 \textsc {I}_{2}^2 \lambda _2+\mu _1 \textsc {I}_{1}+\mu _2 \textsc {I}_{2}},\\&\textsc {E}^{*}= \frac{\mu _1 \textsc {I}_{1}^2 \lambda _1 S+\mu _2 \textsc {I}_{2}^2 \lambda _2 S+\mu _1 \textsc {I}_{1} S+\mu _2 \textsc {I}_{2} S}{\rho _1+\rho _2},\\&\textsc {I}_{1}^{*}=\frac{\textsc {E} \rho _1}{\beta _1+\alpha _1},\\&\textsc {I}_{2}^{*}=\frac{\textsc {E} \rho _2}{\beta _2+\alpha _2},\\&\textsc {R}^{*}=\frac{\beta _1 \textsc {I}_{1}+\beta _2 \textsc {I}_{2}}{\alpha }. \end{aligned}$$

## Basic reproduction number

The virus’s reproduction number, also known as $R_0$, is a way of rating a disease’s propensity to spread and calculate the average number of individuals infected by one infected person. In general, this basic reproduction number $R_{0}$ is an epidemiologic metric used to describe the transmissibility of infectious agents or contagiousness. So in this section, we will utilize the next generation matrix technique to calculate the basic reproductive number for this model. Therefore, the infected compartments of our model are the following:

14 $$\begin{aligned} \left\{ \begin{aligned} ^{\mathrm {C}}{\mathbf {D}}^{\theta }\textsc {E}(\mathrm {t})&=\mu _{1}\textsc {S}(\mathrm {t})\textsc {I}_{1}(\mathrm {t})(1+\lambda _{1}\textsc {I}_{1}(\mathrm {t}))+\mu _{2}\textsc {S}(\mathrm {t})\textsc {I}_{2}(\mathrm {t})(1+\lambda _{2}\textsc {I}_{2}(\mathrm {t}))-(\rho _{1}+\rho _{2})\textsc {E}(\mathrm {t}),\\ ^{\mathrm {C}}{\mathbf {D}}^{\theta }\textsc {I}_{1}(\mathrm {t})&=\rho _{1}\textsc {E}(\mathrm {t})-(\alpha _{1}+\beta _{1})\textsc {I}_{1}(\mathrm {t}),\\ ^{\mathrm {C}}{\mathbf {D}}^{\theta }\textsc {I}_{2}(\mathrm {t})&=\rho _{2}\textsc {E}(\mathrm {t})-(\alpha _{2}+\beta _{2})\textsc {I}_{2}(\mathrm {t}), \end{aligned}\right. \end{aligned}$$

The Jacobian matrix of the system (14) is given by:

$$\begin{aligned} \mathrm {J}_{0}= \begin{pmatrix} -(\rho _{1}+\rho _{2}) &{} \mu _{1}\textsc {S}^{0} &{} \mu _{2}\textsc {S}^{0}\\ \rho _{1} &{} -(\alpha _{1}+\beta _{1}) &{} 0\\ \rho _{2} &{} 0 &{} -(\alpha _{2}+\beta _{2}) \end{pmatrix}. \end{aligned}$$

Now decomposing the matrix $\mathrm {J}_{0}$ in terms of *A* and *B*, that is $\mathrm {J}_{0} = A - B$, we have:

$$\begin{aligned} A= \begin{pmatrix} 0 &{} \mu _{1}\textsc {S}^{0} &{} \mu _{2}\textsc {S}^{0}\\ 0 &{} 0 &{} 0\\ 0 &{} 0 &{} 0 \end{pmatrix},\;\;\; B=\begin{pmatrix} \rho _{1}+\rho _{2} &{} 0 &{} 0\\ -\rho _{1} &{} \alpha _{1}+\beta _{1} &{} 0\\ -\rho _{2} &{} 0 &{} \alpha _{2}+\beta _{2} \end{pmatrix}. \end{aligned}$$

The basic reproduction number is obtained from $R_{0}=\rho (AB^{-1})$, so we have:

15 $$\begin{aligned} R_{0}=\frac{\textsc {S}^{0} (\mu _1 \beta _2 \rho _1+\mu _2 \beta _1 \rho _2+\mu _1 \alpha _2 \rho _1+\mu _2 \alpha _1 \rho _2)}{(\rho _1+\rho _2) (\beta _1+\alpha _1) (\beta _2+\alpha _2)},\end{aligned}$$

where $\textsc {S}^{0}=\frac{\Lambda }{\alpha }.$ Hence, Eq. (15) is the basic reproduction number for the proposed system.

## Stability of equilibrium points

The stability of equilibrium points is investigated in this section. The Jacobian matrix of system (3) is as follows:

$$\begin{aligned} \mathrm {J}= \begin{pmatrix} -\alpha - \mu _1 \textsc {I}_{1}(1+\lambda _1\textsc {I}_{1})-\mu _2 \textsc {I}_{2}(1+\lambda _2\textsc {I}_{2}) &{} 0 &{} -\mu _1 \lambda _1 \textsc {S}\textsc {I}_{1}-\mu _1\textsc {S}(1+\lambda _1\textsc {I}_{1}) &{} -\mu _2 \lambda _2 \textsc {S}\textsc {I}_{2}-\mu _2\textsc {S}(1+\lambda _2\textsc {I}_{2}) &{} 0\\ \mu _1 \textsc {I}_{1}(1+\lambda _1\textsc {I}_{1})+\mu _2 \textsc {I}_{2}(1+\lambda _2\textsc {I}_{2}) &{} -\rho _1-\rho _2 &{} \mu _1 \lambda _1 \textsc {S}\textsc {I}_{1}+\mu _1\textsc {S}(1+\lambda _1\textsc {I}_{1}) &{} \mu _2 \lambda _2 \textsc {S}\textsc {I}_{2}+\mu _2\textsc {S}(1+\lambda _2\textsc {I}_{2}) &{} 0\\ 0 &{} \rho _1 &{} -\alpha _1-\beta _1 &{} 0 &{} 0\\ 0 &{} \rho _2 &{} 0 &{} -\alpha _2-\beta _2 &{} 0 \\ 0 &{} 0 &{} \beta _1 &{} \beta _2 &{} -\alpha \end{pmatrix}. \end{aligned}$$

So, the Jacobian matrix of system at $E^{0}$ is:

$$\begin{aligned} \mathrm {J}(E^{0})= \begin{pmatrix} -\alpha &{} 0 &{} -\frac{\mu _1\Lambda }{\alpha } &{} -\frac{\mu _2\Lambda }{\alpha } &{} 0\\ 0 &{} -\rho _1-\rho _2 &{} \frac{\mu _1\Lambda }{\alpha } &{} \frac{\mu _2\Lambda }{\alpha } &{} 0\\ 0 &{} \rho _1 &{} -\alpha _1-\beta _1 &{} 0 &{} 0\\ 0 &{} \rho _2 &{} 0 &{} -\alpha _2-\beta _2 &{} 0 \\ 0 &{} 0 &{} \beta _1 &{} \beta _2 &{} -\alpha \end{pmatrix}. \end{aligned}$$

### Theorem 8.1

Let $R_{0}$ and $\mathrm {A}_{\mathscr {i}},\;(\mathscr {i} = 1,2,3)$ be defined by (15) and (17), respectively. If $R_{0} <1$, then by Routh–Hurwitz stability criterion [57], the disease-free equilibrium point $E^{0}$ of the model (3) is locally asymptotically stable if and only if $\mathrm {A}_{1}>0, \mathrm {A}_{3}>0$ and $\mathrm {A}_{1}\mathrm {A}_{2}>\mathrm {A}_{3}$.

### Proof

The characteristic equation of the Jacobian matrix at the disease-free equilibrium point is $\det (\mathrm {J}(E^{0})-\phi I)=0$. Then one can achieved the following:

16 $$\begin{aligned} (\phi +\alpha )^{2}(\phi ^{3}+\mathrm {A}_{1}\phi ^{2}+\mathrm {A}_{2}\phi +\mathrm {A}_{3})=0, \end{aligned}$$

where

17 $$\begin{aligned} \left\{ \begin{aligned} \mathrm {A}_{1}&=\beta _1+\beta _2+\alpha _1+\alpha _2+\rho _1+\rho _2,\\ \mathrm {A}_{2}&=(\rho _1+\rho _2)(\beta _1+\alpha _1)-\Lambda \alpha ^{-1}(\mu _1 \rho _1+\mu _2 \rho _2)+(\beta _2+\alpha _2)(\beta _1+\alpha _1)+(\beta _2 +\alpha _2) (\rho _1+\rho _2),\\ \mathrm {A}_{3}&=(\beta _2+\alpha _2)(\rho _1+\rho _2)(\beta _1+\alpha _1)-\Lambda \alpha ^{-1} (\mu _1 \rho _1 (\beta _2+\alpha _2)+\mu _2 \rho _2 (\beta _1+\alpha _1)). \end{aligned}\right. \nonumber \\ \end{aligned}$$

Rewriting the $\mathrm {A}_{3}$ in terms of the basic reproduction number $R_{0}$, $\mathrm {A}_{3}$ becomes:

$$\begin{aligned} \mathrm {A}_{3}&=(\beta _2+\alpha _2)(\rho _1+\rho _2)(\beta _1+\alpha _1)(1-R_{0}). \end{aligned}$$

It is obvious in the first factor of above characteristic Eq. (16) that two eigenvalues $\phi _{1}$ and $\phi _{2}$ are always negative for $\alpha > 0$. The other three eigenvalues can be obtained by solving the following equation:

18 $$\begin{aligned} \phi ^{3}+\mathrm {A}_{1}\phi ^{2}+\mathrm {A}_{2}\phi +\mathrm {A}_{3}=0. \end{aligned}$$

The remaining three eigenvalues have negative real parts, if they follow the Routh–Hurwitz criteria of order 3, such that $\mathrm {A}_{1}>0, \mathrm {A}_{3}>0$ and $\mathrm {A}_{1}\mathrm {A}_{2}>\mathrm {A}_{3}$. Therefore, $R_{0}<1$ if and only if all the eigenvalues have negative real parts. Which completes the proof. $\square$

Individuals who are asymptomatic yet infectious play a critical role in spreading the disease across the population. When $R_{0} > 1$, the endemic equilibrium state of the model (3) is stable, as shown in the following theorem.

### Theorem 8.2

Let $R_{0}$ and $\mathrm {B}_{\mathscr {i}},\;(\mathscr {i} = 1,2,3,4)$ be defined by (15) and (20), respectively. If $R_{0} > 1$, then $\mathrm {B}_{\mathscr {i}} > 0$ for all $\mathscr {i} = 1,2,3,4.$ Then by Routh–Hurwitz stability criterion [57], the endemic equilibrium point $E^{*}$ of the model (3) is locally asymptotically stable if and only if $\mathrm {B}_{1}\mathrm {B}_{2}\mathrm {B}_{3}-\mathrm {B}_{2}^{2}-\mathrm {B}_{1}^{2}\mathrm {B}_{4}>0.$

### Proof

To show the local stability of the proposed model at endemic equilibrium point $E^{*}$, linearizing model (3) about $E^{*}$ such that:

$$\begin{aligned} \mathrm {J}(E^{*})= \begin{pmatrix} -a_1 &{} 0 &{} -a_2 &{} -a_3 &{} 0 \\ a_4 &{} -\left( \rho _1+\rho _2\right) &{} a_2 &{} a_3 &{} 0 \\ 0 &{} \rho _1 &{} -\left( \beta _1+\alpha _1\right) &{} 0 &{} 0 \\ 0 &{} \rho _2 &{} 0 &{} -\left( \beta _2+\alpha _2\right) &{} 0 \\ 0 &{} 0 &{} \beta _1 &{} \beta _2 &{} -\alpha \\ \end{pmatrix}, \end{aligned}$$

where

$$\begin{aligned} a_1&=\alpha +\mu _1 \textsc {I}_{1}^{*} (1+\lambda _1\textsc {I}_{1}^{*})+\mu _2 \textsc {I}_{2}^{*} (1+\lambda _2\textsc {I}_{2}^{*}),\\ a_2&=\mu _1 \textsc {I}_{1}^{*} \lambda _1 \textsc {S}^{*}+\mu _1 \textsc {S}^{*} (1+\lambda _1\textsc {I}_{1}^{*}),\\ a_3&=\mu _2 \textsc {I}_{2}^{*} \lambda _2 \textsc {S}^{*}+\mu _2 \textsc {S}^{*} (1+\lambda _2\textsc {I}_{2}^{*}),\\ a_4&=\mu _1 \textsc {I}_{1}^{*} (1+\lambda _1\textsc {I}_{1}^{*})+\mu _2 \textsc {I}_{2}^{*} (1+\lambda _2\textsc {I}_{2}^{*}). \end{aligned}$$

The characteristic equation associated with $\mathrm {J}(E^{*})$ is:

19 $$\begin{aligned} (-\phi -\alpha )(\phi ^{4}+\mathrm {B}_{1} \phi ^{3}+\mathrm {B}_{2}\phi ^{2}+\mathrm {B}_{3} \phi +\mathrm {B}_{4})=0, \end{aligned}$$

where

20 $$\begin{aligned} \left\{ \begin{aligned} \mathrm {B}_{1}=&\; a_{1}+\beta _{1}+\beta _{2}+\alpha _{1}+\alpha _{2}+\rho _{1}+\rho _{2},\\ \mathrm {B}_{2}=&\; a_1 (\beta _1+\beta _2+\alpha _1+\alpha _2+\rho _1+\rho _2)-a_2 \rho _1-a_3 \rho _2+\beta _1 (\beta _2+\alpha _2+\rho _1+\rho _2)+\beta _2 \alpha _1+\beta _2 \rho _1+\beta _2 \rho _2\\&+\alpha _1 \rho _1+\alpha _1 \rho _2+\alpha _2 \rho _1+\alpha _2 \rho _2+\alpha _2 \alpha _1,\\ \mathrm {B}_{3}=&\; a_2 \rho _1 (a_4-\beta _2-\alpha _2)+a_1 (-a_2 \rho _1-a_3 \rho _2+\beta _2 (\alpha _1+\rho _1+\rho _2)+\beta _1 (\beta _2+\alpha _2+\rho _1+\rho _2)+\alpha _2 \rho _1\\&+\alpha _2 \rho _2+\alpha _1 \rho _1+\alpha _1 \rho _2+\alpha _1 \alpha _2)-a_3 \beta _1 \rho _2-a_3 \alpha _1 \rho _2+a_3 a_4 \rho _2+\beta _2 \alpha _1 \rho _1+\beta _1 \alpha _2 \rho _1+\beta _2 \alpha _1 \rho _2\\ {}&+\beta _1 \alpha _2 \rho _2+\beta _1 \beta _2 \rho _1+\beta _1 \beta _2 \rho _2+\alpha _1 \alpha _2 \rho _1+\alpha _1 \alpha _2 \rho _2,\\ \mathrm {B}_{4}=&\; a_4 (a_2 \rho _1 (\beta _2+\alpha _2)+a_3 \rho _2 (\beta _1+\alpha _1))+a_1 ((\beta _1+\alpha _1) (-a_3 \rho _2+\beta _2 (\rho _1+\rho _2)+\alpha _2 (\rho _1+\rho _2))\\ {}&-a_2 \rho _1 (\beta _2+\alpha _2)). \end{aligned}\right. \end{aligned}$$

From Eq. (19) it is clear that there are five corresponding eigenvalues of $\mathrm {J}(E^{*})$. One of them is $\phi _{1} = -\alpha$, having negative real part. The remaining four can be obtained by the solution of the following equation:

21 $$\begin{aligned} \phi ^{4}+\mathrm {B}_{1} \phi ^{3}+\mathrm {B}_{2}\phi ^{2}+\mathrm {B}_{3}\phi +\mathrm {B}_{4}=0, \end{aligned}$$

Using Routh–Hurwitz criterion of order 4, we can conclude that given system (3) is local asymptotic stable at $E^{*}$. $\square$

## $R_{0}$ Sensitivity analysis

The sensitivity analysis is applied to study the effect of the parameters on proposed COVID-19 outbreak model. In particular, it is necessary to identify the most sensitive parameters causing a disturbance in model dynamics by a small change in numeric value. To check the $R_{0}$ sensitivity, we calculate its derivatives as follows:

$$\begin{aligned}&\frac{\partial R_{0}}{\partial \Lambda }=\frac{\mu _1 \beta _2 \rho _1+\mu _2 \beta _1 \rho _2+\mu _1 \alpha _2 \rho _1+\mu _2 \alpha _1 \rho _2}{\alpha (\rho _1+\rho _2) (\beta _1+\alpha _1) (\beta _2+\alpha _2)},\\&\frac{\partial R_{0}}{\partial \alpha }=-\frac{\Lambda (\mu _1 \beta _2 \rho _1+\mu _2 \beta _1 \rho _2+\mu _1 \alpha _2 \rho _1+\mu _2 \alpha _1 \rho _2)}{\alpha ^{2} (\rho _1+\rho _2) (\beta _1+\alpha _1) (\beta _2+\alpha _2)},\\&\frac{\partial R_{0}}{\partial \mu _{1}}=\frac{\Lambda \rho _1}{\alpha (\rho _1+\rho _2) (\beta _1+\alpha _1)},\\&\frac{\partial R_{0}}{\partial \mu _{2}}=\frac{\Lambda \rho _2}{\alpha (\rho _1+\rho _2) (\beta _2+\alpha _2)},\\&\frac{\partial R_{0}}{\partial \rho _{1}}=-\frac{\Lambda \rho _2 (\mu _2 (\beta _1+\alpha _1)-\mu _1 (\beta _2+\alpha _2))}{\alpha (\rho _1+\rho _2)^2 (\beta _1+\alpha _1) (\beta _2+\alpha _2)},\\&\frac{\partial R_{0}}{\partial \rho _{2}}=\frac{\Lambda \rho _1 (\mu _2 (\beta _1+\alpha _1)-\mu _1 (\beta _2+\alpha _2))}{\alpha (\rho _1+\rho _2)^2 (\beta _1+\alpha _1) (\beta _2+\alpha _2)},\\&\frac{\partial R_{0}}{\partial \alpha _{1}}=-\frac{\mu _1 \Lambda \rho _1}{\alpha (\rho _1+\rho _2) (\beta _1+\alpha _1)^2},\\&\frac{\partial R_{0}}{\partial \alpha _{2}}=-\frac{\mu _2 \Lambda \rho _2}{\alpha (\rho _1+\rho _2) (\beta _2+\alpha _2)^2},\\&\frac{\partial R_{0}}{\partial \beta _{1}}=-\frac{\mu _1 \Lambda \rho _1}{\alpha (\rho _1+\rho _2) (\beta _1+\alpha _1)^2},\\&\frac{\partial R_{0}}{\partial \beta _{2}}=-\frac{\mu _2 \Lambda \rho _2}{\alpha (\rho _1+\rho _2) (\beta _2+\alpha _2)^2}. \end{aligned}$$

Since, all the parameters are positive so $\frac{\partial R_{0}}{\partial \Lambda }, \frac{\partial R_{0}}{\partial \mu _{1}}, \frac{\partial R_{0}}{\partial \mu _{2}}, \frac{\partial R_{0}}{\partial \rho _{2}}>0$. It concludes that the reproduction number ($R_{0}$) is increasing with $\Lambda , \mu _{1}, \mu _{2}$ and $\rho _{2}$. The normalized sensitivity indices corresponding to these parameters are estimated as follows:

$$\begin{aligned}&\Gamma _{\Lambda }=\frac{\Lambda }{R_{0}}\frac{\partial R_{0}}{\partial \Lambda }=1,\\&\Gamma _{\mu _{1}}=\frac{\mu _{1}}{R_{0}}\frac{\partial R_{0}}{\partial \mu _{1}}=\frac{\mu _1 \rho _1 (\beta _2+\alpha _2)}{\mu _1 \rho _1 (\beta _2+\alpha _2)+\mu _2 \rho _2 (\beta _1+\alpha _1)},\\&\Gamma _{\mu _{2}}=\frac{\mu _{2}}{R_{0}}\frac{\partial R_{0}}{\partial \mu _{2}}=\frac{\mu _2 \rho _2 (\beta _1+\alpha _1)}{\mu _1 \rho _1 (\beta _2+\alpha _2)+\mu _2 \rho _2 (\beta _1+\alpha _1)},\\&\Gamma _{\rho _{2}}=\frac{\rho _{2}}{R_{0}}\frac{\partial R_{0}}{\partial \rho _{2}}=\frac{\rho _1 \rho _2 (\mu _2 (\beta _1+\alpha _1)-\mu _1 (\beta _2+\alpha _2))}{(\rho _1+\rho _2) (\mu _1 \rho _1 (\beta _2+\alpha _2)+\mu _2 \rho _2 (\beta _1+\alpha _1))}. \end{aligned}$$

**Fig. 2:**
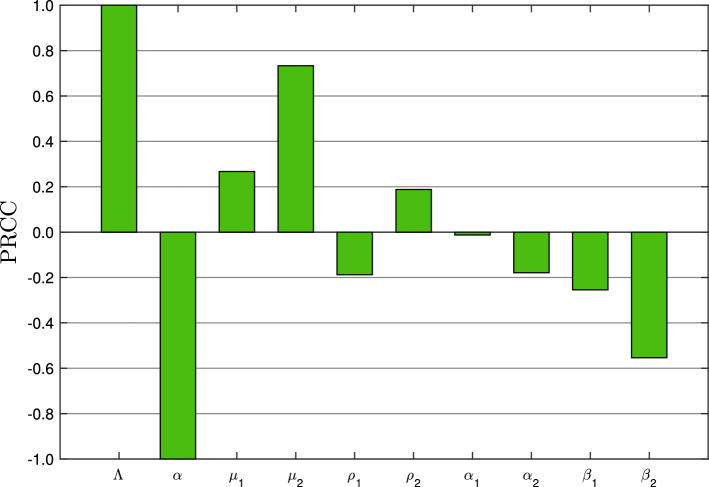
PRCC results for significance of parameters involved in $R_{0}$

Here, the sensitivity index can be constant depending on some parameters or can be free of any independent parameters. The partial rank correlation coefficient (PRCC) results for significance of parameters involved in $R_{0}$ can be seen in Fig. 2 and in Table 2.

## Numerical results and discussion

In this section, we discuss the impact of parameters on reproduction number $R_{0}$ in numerical simulations of the proposed model using the fractional Euler method [58]. The numerical simulation of the present model (3) will be carried out to support the analytical result using the value of the parameters as given in Table 1.

**Table 1:** List of used parameters in this study

Parameters	Values/Day	Source
$\Lambda$	$4.21\times 10^{-05}$	[59]
$\alpha$	$4.21\times 10^{-05}$	[59]
$\mu _{1}$	0.2516	Assumed
$\mu _{2}$	0.3110	[60]
$\lambda _{1}$	0.2213	Assumed
$\lambda _{2}$	0.3041	Assumed
$\rho _{1}$	0.1516	Assumed
$\rho _{2}$	0.1818	[60]
$\beta _{1}$	0.0700	Assumed
$\beta _{2}$	0.0300	Assumed
$\alpha _{1}$	$3.50\times 10^{-03}$	Assumed
$\alpha _{2}$	$9.70\times 10^{-03}$	[60]

The sensitivity analysis is carried out by estimating the sensitivity indices based on the parameter values to examine the parameter impact on $R_{0}$. The sensitivity indices given in Table 2 are obtained by using the values of involved parameters. From the computed sensitivity indices, it can be seen that a $10\%$ increase in the recruitment rate $\Lambda$, asymptomatic transmission rate $\mu _1$, symptomatic transmission rate $\mu _2$, and symptomatic infection rate $\rho _{2}$ cause to increase the value of $R_0$ by $10\%$, $2.6\%$, $7.3\%$, and $1.8\%$, respectively, and can lead to an outbreak subsequently. However, it can be noticed that the symptomatic infection rate $\rho _{2}$ does not affect $R_0$ significantly. But on the other hand, asymptomatic infection rate $\rho _{1}$, asymptomatic recovery rate $\beta _{1}$, symptomatic recovery rate $\beta _{2}$, asymptomatic death rate $\alpha _{1}$ and symptomatic death rate $\alpha _{2}$ describe that increasing their values by $10\%$ will decrease the value of $R_{0}$ by $1.8\%$, $2.5\%$, $5.5\%$, $0.1\%$, and $1.7\%$, respectively.

**Table 2:** Sensitivity indices of the $R_{0}$ against the parameters

Sensitivity index	Value	Sensitivity index	Value
$\Gamma _\Lambda$	1.00000	$\Gamma _{\rho _{2}}$	0.18764
$\Gamma _\alpha$	$-1.00000$	$\Gamma _{\alpha _{1}}$	$-0.01271$
$\Gamma _{\mu _{1}}$	0.26706	$\Gamma _{\alpha _{2}}$	$-0.17907$
$\Gamma _{\mu _{2}}$	0.73293	$\Gamma _{\beta _{1}}$	$-0.25435$
$\Gamma _{\rho _{1}}$	$-0.18764$	$\Gamma _{\beta _{2}}$	$-0.55385$

**Fig. 3:**
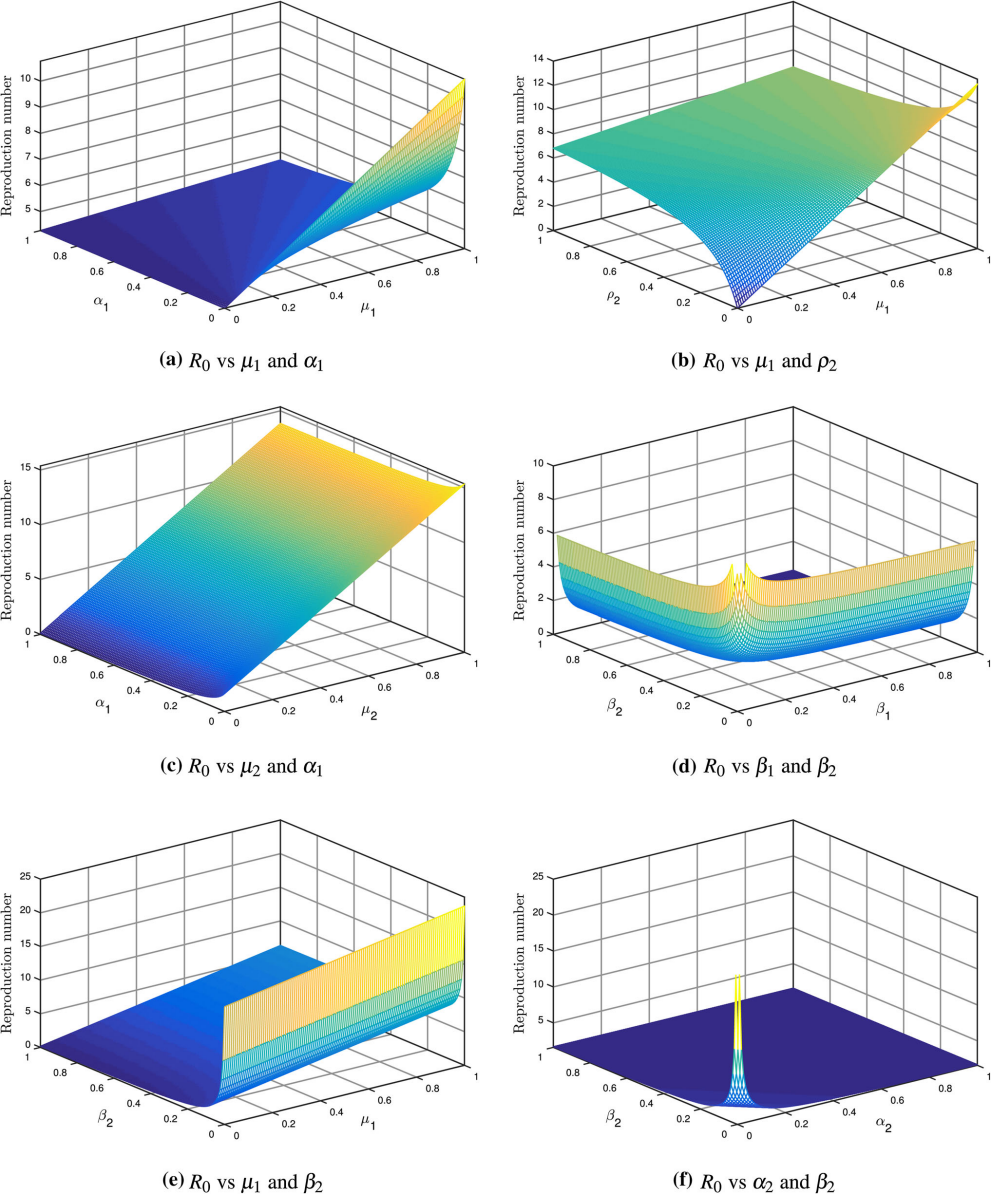
The behavior of $R_{0}$ with different parameters

The impact of some parameters on the reproduction number is shown in Fig. 3. Figure 3a describes the reproduction number $R_{0}$ sensitivity versus the asymptomatic transmission rate $\mu _{1}$ and asymptomatic death rate $\alpha _{1}$. Since from Table 2, it is clear that $\mu _{1}$ has a positive impact while $\alpha _{1}$ has a negative impact on reproduction number $R_{0}$. So, it can be seen in Fig. 3a the asymptomatic death rate has no impact on $R_{0}$ because no variation can be seen in $R_{0}$ when $\alpha _{1}$ increases. On another side, the asymptomatic transmission rate $\mu _{1}$ has a very high impact on $R_{0}$. Figure 3b describes the reproduction number $R_{0}$ sensitivity versus symptomatic infection rate $\rho _{2}$ and asymptomatic transmission rate $\mu _{1}$. So it can be seen in Table 2 that both parameters has a positive impact on reproduction number $R_{0}$. But the impact of asymptomatic transmission rate is higher than the symptomatic infection rate, which is depicted in Fig. 3b.

**Fig. 4:**
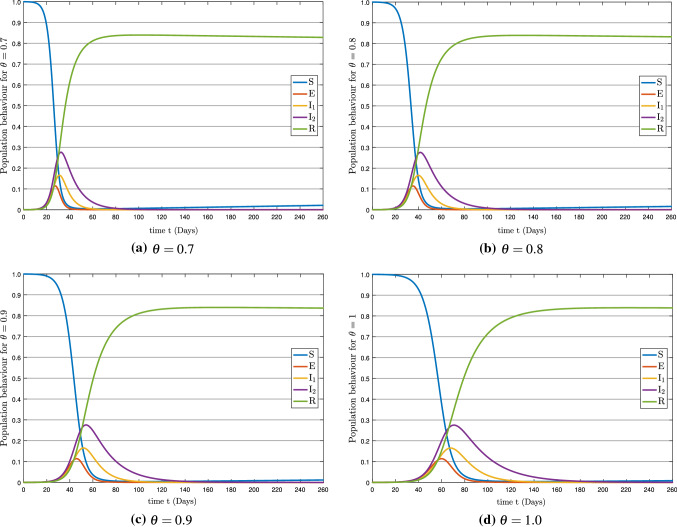
The solution behavior of the all compartments for various values of $\theta$

Figure 3c shows the reproduction number $R_{0}$ sensitivity versus the symptomatic transmission rate $\mu _{2}$ and asymptomatic death rate $\alpha _{1}$. The impact of the symptomatic transmission rate is positive on the reproduction number, while the impact of the asymptomatic death rate is negative. The impact of asymptomatic death rate is almost negligible, but the effect of the symptomatic transmission rate is quite high enough where can be seen in Fig. 3c. Figure 3d shows the reproduction number $R_{0}$ sensitivity versus asymptomatic recovery rate $\beta _{1}$ and symptomatic recovery rate $\beta _{2}$. Both parameters have a negative impact on the reproduction number $R_{0}$ (see Table 2), but the affect is almost the same, which is shown in Fig. 3d.

Figure 3e demonstrates the reproduction number $R_{0}$ sensitivity versus the asymptomatic transmission rate $\mu _{1}$ and symptomatic recovery rate $\beta _{2}$. The transmission rate $\mu _{1}$ of the asymptomatic pathway has a positive impact on reproduction number $R_{0}$ and symptomatic recovery rate $\beta _{2}$ has a negative impact on $R_{0}$, which can be seen in Table 2. From Fig. 3e, we can conclude that the symptomatic transmission rate $\mu _{1}$ effects the reproduction number more than the symptomatic recovery rate $\beta _{2}$. Figure 3f describes the reproduction number $R_{0}$ sensitivity versus symptomatic death rate $\alpha _{2}$ and symptomatic recovery rate $\beta _{2}$. Both parameters have a negative impact on reproduction number $R_{0}$. But the impact is almost the same, which is shown in Fig. 3f.

**Fig. 5:**
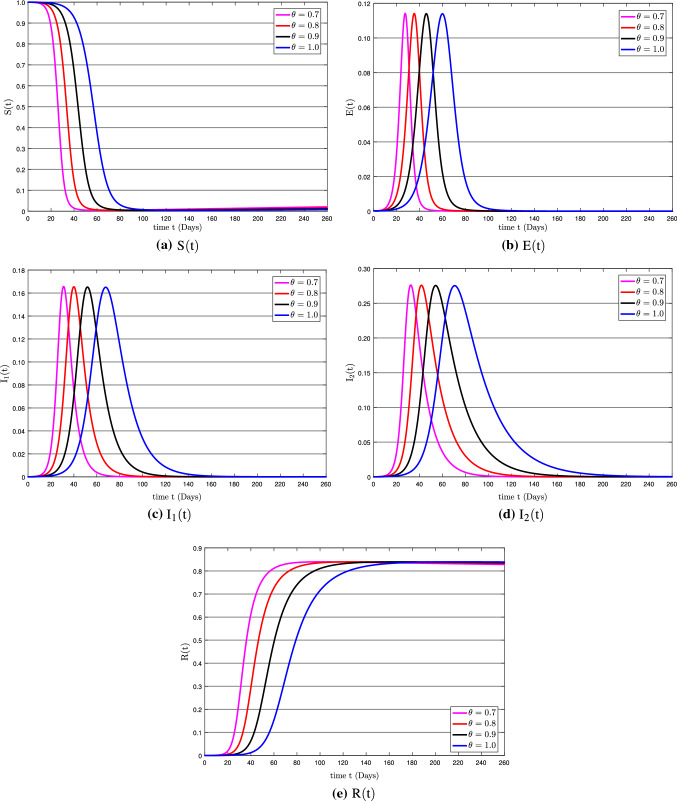
The solution behavior of $\textsc {S}(\mathrm {t}),\; \textsc {E}(\mathrm {t}),\; \textsc {I}_{1}(\mathrm {t}),\; \textsc {I}_{2}(\mathrm {t})$ and $\textsc {R}(\mathrm {t})$ under different fractional order

Now we discuss the numerical results of the governing model in terms of approximate solutions. To achieve this goal, we used the efficient fractional-order Euler technique with the Caputo fractional operator. The initial conditions are considered as $\textsc {S}(0) = 1-\textsc {E}(0)-\textsc {I}_{1}(0)-\textsc {I}_{2}(0)-\textsc {R}(0),\; \textsc {E}(0) = 5/20000,\; \textsc {I}_{1}(0) = 0,\; \textsc {I}_{2}(0) = 0,\; \textsc {R}(0) = 0$ with the total population $\textsc {N}=1$ and the values of the parameter are given in Table 1. We have utilized the MATLAB software to simulate the numerical results. Graphs are depicted the approximate solutions obtained by using the suggested numerical approaches under the value of the considered parameters. The dynamics of the suggested model population are graphically shown in Fig. 4 using the Caputo fractional derivative with time sequence framework. Figure 4a shows the population behavior for $\theta =0.7$, Fig. 4b shows the population behavior for $\theta =0.8$, Fig. 4c shows the population behavior for $\theta =0.9$ and the population behavior for $\theta =1.0$ is shown in Fig. 4d.

Figure 5 shows the solution behavior of $\textsc {S}(\mathrm {t}),\; \textsc {E}(\mathrm {t}),\; \textsc {I}_{1}(\mathrm {t}),\; \textsc {I}_{2}(\mathrm {t})$, and $\textsc {R}(\mathrm {t})$ for $\theta =0.7, 0.8, 0.9, 1.0$. Figure 5a shows that the susceptible population decreases rapidly at different rates and stabilizes after about 100 days due to restriction of individual movements. As we can see that the decay rate is faster for a small value of $\theta$, since the susceptible population $\textsc {S}(\mathrm {t})$ is decreasing, which affects the exposed population $\textsc {E}(\mathrm {t})$ and more individuals get the infection. That is why we see an increase in the exposed population in Fig. 5b. Besides, the exposed population $\textsc {E}(\mathrm {t})$ in Fig. 5b is increased very quickly after 15 days and a decrease occurs almost after 70 days and then stables after 110 days. This decrease is happened due to the imposition of movement restrictions. Moreover, the exposed population increases and decreases rapidly for smaller values of $\theta$ as compared to bigger values.

The asymptomatic class $\textsc {I}_{1}(\mathrm {t})$ and symptomatic class $\textsc {I}_{2}(\mathrm {t})$ in Fig. 5c and  d have almost the same pattern, but the asymptomatic population becomes stable after 150 days and the symptomatic population becomes stable after 200 days. Both populations have grown rapidly, but the decline occurred due to standard operating procedures (SOPs) and vaccinations. The infection increases and decreases quickly for smaller values of $\theta$. Since we know that many innocent people have been died due to this pandemic, but there are many who have survived as well due to timely treatment and following the SOPs. Most people have been vaccinated and are now protected from infection. 52.9% population have received their first dose of the COVID-19 vaccination worldwide till November 13, 2021 [61], which reduced the infection and that is why the recovery rate has almost been stable. So in Fig. 5e, it can be seen that the recovery rate is almost 83% of the population. The increase in recovery is due to a strong protection rate, people’s awareness about the infection, government action, and vaccination. Besides, this rise up of recovery is also different for various values of fractional order.

**Fig. 6:**
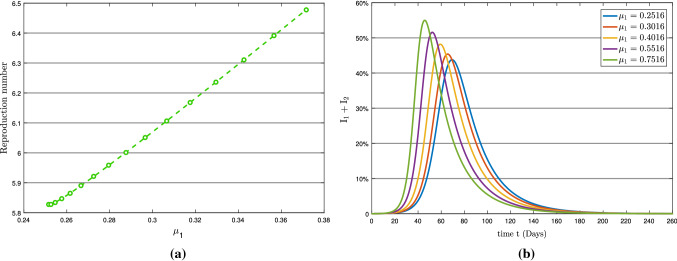
(**a**) Reproduction number with respect to $\mu _{1}$ and (**b**) Variation in total infection

**Fig. 7:**
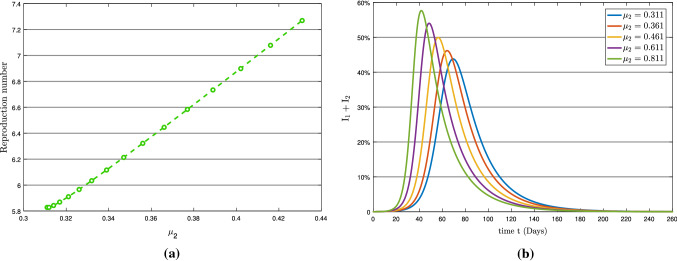
(**a**) Reproduction number with respect to $\mu _{2}$ and (**b**) Variation in total infection

**Fig. 8:**
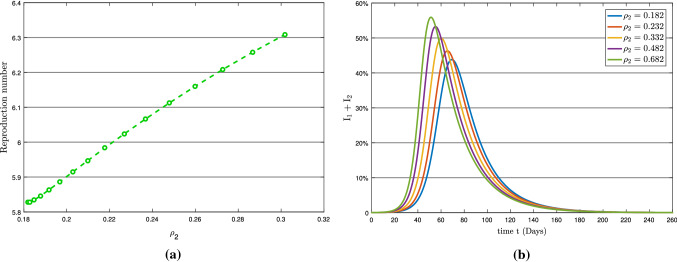
(**a**) Reproduction number with respect to $\rho _{2}$ and (**b**) Variation in total infection

**Fig. 9:**
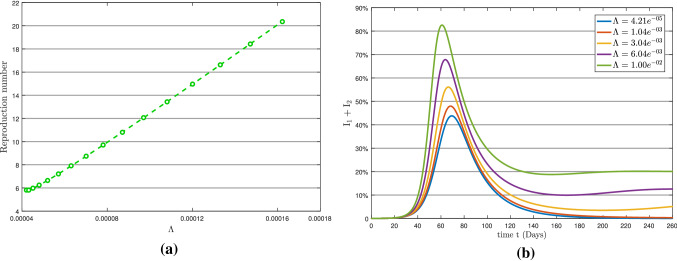
(**a**) Reproduction number with respect to $\Lambda$ and (**b**) Variation in total infection

**Fig. 10:**
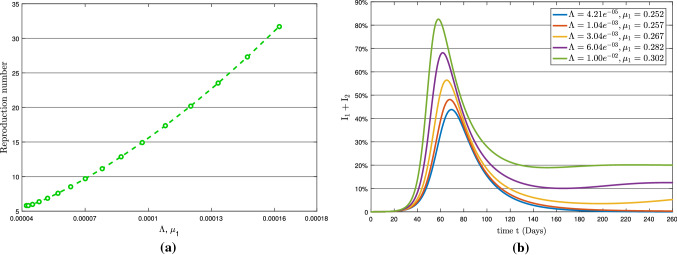
(**a**) Reproduction number with respect to $\Lambda$ and $\mu _{1}$ and (**b**) Variation in total infection

**Fig. 11:**
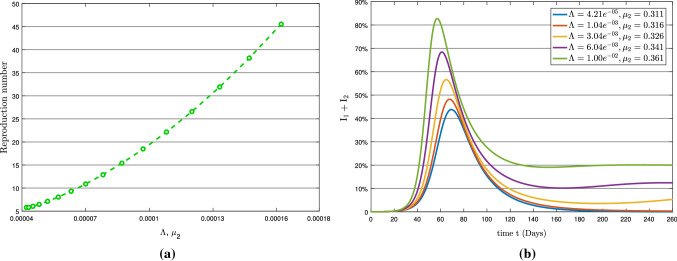
(**a**) Reproduction number with respect to $\Lambda$ and $\mu _{2}$ and (**b**) Variation in total infection

Here, we discuss the impact of the parameters $\Lambda , \mu _{1}, \mu _{2}$ and $\rho _{2}$ on the basic reproduction number $R_{0}$, where the values of mentioned parameters is given in Table 2. The sensitive indices (mentioned above parameters) will be used to assess the propensity of the total infection ($\textsc {I}_{1}+\textsc {I}_{2}$) curve. Table 3 shows the loop iterations used to simulate model (3). The simulation has used different sensitive parameter values to generate graphs of total infection and basic reproduction number $R_{0}$ to examine the COVID-19 epidemic pattern while the other parameters remain unchanged. The graphs are displayed via a panel in Figs. 6, 7, 8, 9, 10, 11, where the left showing the basic reproduction number pattern and the right indicating the total infection behavior concerning a specific sensitive parameter.

**Table 3:** Loop iterations corresponding to the sensitive parameters

Parameters	Loop iteration		Type of effect
	Total Infection Plot	Reproduction Number Plot	
	$(1\le {\mathscr {i}}\le 5)$	$(1\le {\mathscr {i}}\le 15)$	
$\mu _{1}$	$\mu _{1}+{\mathscr {i}}\times 0.05$	$\mu _{1}+{\mathscr {i}}\times 0.001$	Individual
$\mu _{2}$	$\mu _{2}+{\mathscr {i}}\times 0.05$	$\mu _{2}+{\mathscr {i}}\times 0.001$	Individual
$\rho _{2}$	$\rho _{2}+{\mathscr {i}}\times 0.05$	$\rho _{2}+{\mathscr {i}}\times 0.001$	Individual
$\Lambda$	$\Lambda +\mathscr {i}\times 0.001$	$\Lambda +\mathscr {i}\times 0.000001$	Individual
$\mu _{1},\Lambda$	$\mu _{1}+{\mathscr {i}}\times 0.005$	$\mu _{1}+{\mathscr {i}}\times 0.005$	Combined effect
	$\Lambda +{\mathscr {i}}\times 0.001$	$\Lambda +{\mathscr {i}}\times 0.000001$	of $\mu _{1}$ and $\Lambda$
$\mu _{2},\Lambda$	$\mu _{2}+{\mathscr {i}}\times 0.005$	$\mu _{2}+{\mathscr {i}}\times 0.005$	Combined effect
	$\Lambda +{\mathscr {i}}\times 0.001$	$\Lambda +{\mathscr {i}}\times 0.000001$	of $\mu _{2}$ and $\Lambda$

According to the sensitivity analysis, the symptomatic transmission rate $\mu _{2}$ is the second most sensitive component impacting the epidemic. The parameters $\mu _{1}$ and $\mu _{2}$ have nearly a similar effect on basic reproduction number $R_{0}$. In comparison with asymptomatic transmission rate $\mu _{1}$ in Fig. 6a, with symptomatic transmission rate $\mu _{2}$ in Fig. 7a, $\mu _{2}$ causes a quicker and higher increase in the total infection. The graph in Fig. 7b shows that roughly 58 percent of the overall population becomes infected with the symptomatic transmission rate $\mu _{2}$ on day 40 (approximately). In contrast, about 52 percent of the total population becomes infected with asymptomatic transmission rate $\mu _{1}$ on day 48 in Fig. 6b. Consequently, maintaining self-isolation and quarantine would be the most effective ways to control COVID-19, which is disseminated by symptomatic transmission.

Figure 8 depicts the effect of the symptomatic infection rate $\rho _{2}$ on basic reproduction number $R_{0}$ and on total infection. From Table 3 and Fig. 8a, it can be seen that the symptomatic infection rate $\rho _{2}$ is less sensitive to the $R_0$. Moreover, as shown in Fig. 8b, it has a significant impact on the total infection of COVID-19 pandemic. Figure 9b shows the high effect of recruitment rate $\Lambda$ on $R_0$. Also, it is shown for a minimal increase in $\Lambda$, the total number of infected people increases significantly.

Imposing a ban on population migrating from one place to another would thus be the most effective way of handling this outbreak. The initial strategic action policy should be to established a management plan to control $\Lambda$ in order to keep $R_0<1$. As it is shown in Fig. 9a, the value of $R_0$ reaches from 5.8 to 20 for a minimal change in $\Lambda$ from 0.00004 to 0.00016, indicating that the recruitment rate to a region during the transmission of COVID-19 has a significant impact on the pandemic.

Furthermore, Figs. 10 and 11 present the simultaneous impact of $(\Lambda , \mu _{\mathscr {i}}),\;(\mathscr {i}=1,2)$ on basic reproduction number and total infected population. Figure 10 illustrates the impact of $(\Lambda , \mu _{1})$ on $R_{0}$ and the total infected population while Fig. 11 demonstrates the impact of $(\Lambda , \mu _{2})$. Both total infection graphs 10b and 11b are almost identical but $(\Lambda , \mu _{2})$ significantly impacts basic reproduction number compared to $(\Lambda , \mu _{1})$. This means that the severe endemic will happen if the recruitment rate and symptomatic transmission rate change simultaneously.

**Fig. 12:**
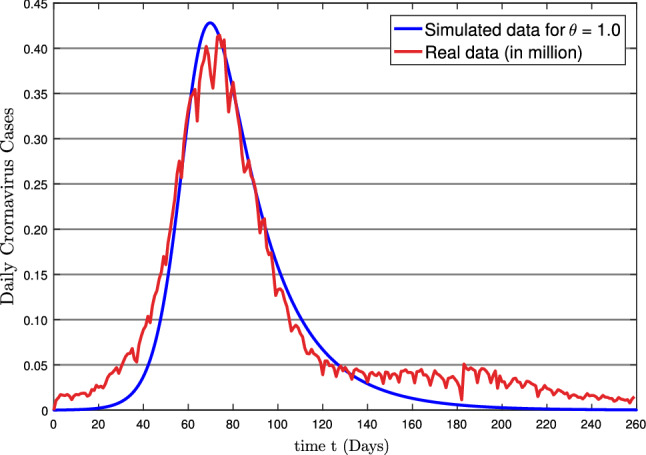
Real data vs. Simulated data for the second wave of COVID-19 in India from 22 February 2021 to 10 November 2021

Recently, India has faced the second wave of the COVID-19 pandemic, which had serious implications in the form of spiraling cases, restricted availability of essential medications, and increased mortality, particularly among the young population. After the second wave, the country was infected approximately by 34.4 million individuals and about 0.4 million died innocent people as of November 13, 2021. The second wave was spread rapidly and had severely impacted the whole country. It is thought to be the second worst epidemic to hit India nearly after a century. The last menace occurred during the 1918 influenza outbreak, which claimed the lives of millions people. Here, we have taken the real data of COVID-19 spread in India from February 22, 2021, until November 10, 2021, from reference [62] and compared it with the simulated data in this research, which is shown in Fig. 12.

## Conclusion

This study was aimed to investigate the effect of symptomatic and asymptomatic transmissions on the coronavirus (COVID-19) outbreak using a fractional-order mathematical model, which is achieved successfully. The existence theory and uniqueness of proposed model solution have been carried out with the help of Schaefer- and Banach-type fixed point theorems. The local stability of the model is established through the Routh–Hurwitz criteria, which is concluded that the disease-free equilibrium is stable for $R_{0} < 1,$ whereas the endemic equilibrium becomes stable for $R_{0} > 1$ and unstable otherwise. The effect of the various parameters on the reproduction number $(R_{0})$ has also been analyzed. Besides, the transmission of the disease under various fractional orders is investigated. In Fig. 11, it can be seen that the recruitment rate and symptomatic transmission rate are significantly impacting the reproduction number. Therefore, the COVID-19 epidemic can be significantly reduced by controlling people from migrating and strictly enforcing personal measures simultaneously. The total infected population plot has been compared with the real infected population of India’s second wave of COVID-19 outbreak (proportion of the population) in Fig. 12 which showed the great agreement of simulated results in this research with the real data. It is worth mentioning that the proposed model here can be studied under stochastic or fuzzy differential operators in future research.

## Data Availability

This manuscript has associated data in a data repository. [The datasets analysed during the current study are available in the references [59] and [60]. Some of used parameters listed in Table 1 are assumed in this study].
